# Prions: Current Progress in Advanced Research

**DOI:** 10.3201/eid2001.131361

**Published:** 2014-01

**Authors:** Ryan A. Maddox

**Affiliations:** Affiliation: Centers for Disease Control and Prevention, Atlanta, Georgia, USA

**Keywords:** prion, spongiform encephalopathy, Creutzfeldt-Jakob disease

This 134-page book provides a brief but wide-ranging overview of current research into prions, the infectious proteinaceous agents believed to be responsible for a range of fatal, neurodegenerative diseases including “mad cow” disease and Creutzfeldt-Jakob disease. Divided into 11 chapters, the book covers topics such as prion biology, disease pathogenesis, and strategies for vaccination and treatment. The history and current status of animal prion diseases, including bovine spongiform encephalopathy (BSE), chronic wasting disease (CWD), and scrapie, are also described.

The book delivers on its title of “current progress,” providing information on a range of recent developments, including diagnostic advances such as real-time quaking-induced conversion, and novel disease descriptions including variably protease sensitive prionopathy. The sections on atypical BSE are particularly enlightening and emphasize that, while the incidence of classical BSE may be decreasing, surveillance and research are still essential.

The format of the book leads to inevitable redundancies: the authors of different chapters provide similar background information. More distracting, however, is the occasionally imperfect English; although the reader can usually determine the author’s intent, grammatical errors can make already abstruse subject matter even more difficult to comprehend. Additionally, the book’s breadth, although generally a strength, results in an oversimplification of certain topic areas. A reader with limited knowledge of prion diseases, for example, could get the impression that CWD readily transmits to cattle and goats, because the text states that these animals are “CWD-susceptible” without clarifying that such transmissions have only been achieved through intracerebral inoculation.

Prions: Current Progress in Advanced Research is an excellent introduction to the many facets of these unusual agents and the challenges confronted by those studying them. I recommend it to students and researchers wishing to gain some insight into this unique field. However, supplementation with peer-reviewed prion literature is also warranted, as both a means to obtain additional background information and as a way to remain informed about rapidly developing scientific advances.

